# A screen for MeCP2-TBL1 interaction inhibitors using a luminescence-based assay

**DOI:** 10.1038/s41598-023-29915-z

**Published:** 2023-03-08

**Authors:** Beatrice Alexander-Howden, Li Zhang, Almer M. van der Sloot, Sylvain Tollis, Daniel J. St-Cyr, Frank Sicheri, Adrian P. Bird, Mike Tyers, Matthew J. Lyst

**Affiliations:** 1 Wellcome Centre for Cell Biology, University of Edinburgh, Michael Swann Building, Max Born Crescent, Edinburgh, EH9 3BF UK; 2 Institute for Research in Immunology and Cancer (IRIC), Department of Medicine, Université de Montréal, Montréal, Québec H3T 1J4 Canada; 3 Mila – Quebec Artificial Intelligence Institute, 6666 Rue Saint-Urbain, Montréal, QC H2S 3H1 Canada; 4 Institute of Biomedicine, University of Eastern Finland, 70210 Kuopio, Finland; 5 X-Chem Inc, 7171 Frederick-Banting, Montréal, QC H4S 1Z9 Canada; 6 Lunenfeld-Tanenbaum Research Institute, Sinai Health System, Toronto, ON M5G 1X5 Canada; 7 The Hospital for Sick Children Research Institute, Toronto, ON M5G 0A4 Canada; 8 Department of Molecular Genetics, University of Toronto, Toronto, ON M5S 1A8 Canada

**Keywords:** Biochemistry, Biological techniques, Drug discovery, Molecular biology, Neuroscience

## Abstract

Understanding the molecular pathology of neurodevelopmental disorders should aid the development of therapies for these conditions. In MeCP2 duplication syndrome (MDS)—a severe autism spectrum disorder—neuronal dysfunction is caused by increased levels of MeCP2. MeCP2 is a nuclear protein that binds to methylated DNA and recruits the nuclear co-repressor (NCoR) complex to chromatin via an interaction with the WD repeat-containing proteins TBL1 and TBLR1. The peptide motif in MeCP2 that binds to TBL1/TBLR1 is essential for the toxicity of excess MeCP2 in animal models of MDS, suggesting that small molecules capable of disrupting this interaction might be useful therapeutically. To facilitate the search for such compounds, we devised a simple and scalable NanoLuc luciferase complementation assay for measuring the interaction of MeCP2 with TBL1/TBLR1. The assay allowed excellent separation between positive and negative controls, and had low signal variance (Z-factor = 0.85). We interrogated compound libraries using this assay in combination with a counter-screen based on luciferase complementation by the two subunits of protein kinase A (PKA). Using this dual screening approach, we identified candidate inhibitors of the interaction between MeCP2 and TBL1/TBLR1. This work demonstrates the feasibility of future screens of large compound collections, which we anticipate will enable the development of small molecule therapeutics to ameliorate MDS.

## Introduction

Rett syndrome-related disorders are severe neurological syndromes associated with dysfunction of the X-linked methyl CpG binding protein 2 (*MECP2*) gene. Loss-of-function mutations in *MECP2* underlie Rett syndrome (RTT)^1^, a disorder which affects approximately 1 in 10,000 females. A defining feature of RTT is a 6–18 month period of apparently normal development. After this time, symptoms appear, and they typically include breathing abnormalities, severe intellectual disability, loss of speech, and the substitution of purposeful hand use with repetitive stereotypies^2–5^. Gain-of-function in MeCP2 is also associated with severe neurological disease^6,7^, with MeCP2 duplication syndrome (MDS) accounting for approximately 1% of X-linked intellectual disability in males^8^. While some features of MDS overlap with those of RTT, the disorder is distinct and is also frequently characterized by neonatal hypotonia, drug-resistant epilepsy and premature death^9,10^.

MeCP2 was first identified in a search for proteins with specific affinity for DNA methylated at CpG dinucleotides^11^. Due to its status as an epigenetic reader protein, and its role in neurological disease, MeCP2 became the subject of intense study. A view emerged of MeCP2 as an abundant nuclear protein which binds broadly across the genome^12,13^ at sites of cytosine modification not restricted to CpG methylation^14–18^. MeCP2 binding then results in slight transcriptional repression of thousands of genes, which tend to be both long and enriched for MeCP2 binding sites^14,18–22^. This transcriptional repression is mediated by MeCP2 recruitment of the nuclear co-repressor (NCoR) complex^23,24^ to DNA via an interaction with the WD repeat-containing NCoR subunits transducin β-like protein 1 (TBL1, a.k.a. TBL1X) and TBL1-Related protein (TBLR1, a.k.a. TBL1XR1)^25,26^. The interaction between the NID (NCoR interaction domain) of MeCP2 and the WD repeat domains of TBL1/TBLR1 is abolished by a cluster of RTT-causing missense mutations in MeCP2^25^, suggesting that failure of MeCP2 to recruit NCoR to chromatin is a key aspect of the pathology of RTT^27,28^.

The toxicity of excess MeCP2 in mice depends on the short peptide motif in MeCP2 which interacts with the WD repeat domains of TBL1/TBLR1^29,30^. This observation—that MDS pathology is dependent on the interaction between MeCP2 and TBL1/TBLR1—raises the possibility of pharmacologically modulating this interaction as a therapeutic strategy. This therapeutic approach is also supported by the following observations: Firstly, normalization of MeCP2 levels in a mouse model of MDS results in a reversal of the neurological deficits present in adult animals^31^. This demonstrates that, as is the case with the pathology of RTT^32^, the damage in the brain associated with MDS is not irreparable, and so an appropriate pharmacological intervention would be expected to have a therapeutic benefit. Secondly, WD repeat domain mediated protein–protein interactions have emerged recently as potential drug targets^33^. WD repeat domain interactions that have been successfully targeted with small molecules include those of the Cdc4 substrate recognition subunit of the SCF ubiquitin ligase complex^34^, the polycomb protein EED^35^, and the MLL/SET complex component WDR5^36^. Thirdly, we have previously solved the structure of the WD repeat domain of TBLR1 in complex with an MeCP2-derived peptide that encompasses the binding motif^26^. The availability of this structure should facilitate the future use of medicinal chemistry to develop hit compounds identified in small molecule library screens into more potent interaction inhibitors.

Here, we set out to develop methods to facilitate the identification of small molecules that perturb the interaction between MeCP2 and the WD repeat domains of TBL1/TBLR1. To enable high throughput screening of compound libraries and the discovery of small molecules capable of inhibiting the association between MeCP2 and TBL1/TBLR1, we have devised a simple and scalable assay for measuring this interaction. The assay is based on protein-fragment complementation of the small and bright luciferase enzyme NanoLuc^37,38^. Using this assay in conjunction with a counter screen based on NanoLuc complementation by the two subunits of protein kinase A (PKA) allows for the elimination of false positives arising due to direct effects on NanoLuc activity. Screening of compound libraries with these parallel NanoLuc assays allowed us to identify candidate inhibitors of the association between MeCP2 and TBL1/TBLR1. This proof-of-principle work demonstrates the feasibility of future screens of large collections of compounds that should uncover more potent inhibitors. We suggest that this approach is a promising strategy for the development of a pharmacological treatment for MDS.

## Results

### A NanoLuc-based protein-fragment complementation assay for MeCP2-TBL1 binding

**Figure 1:**
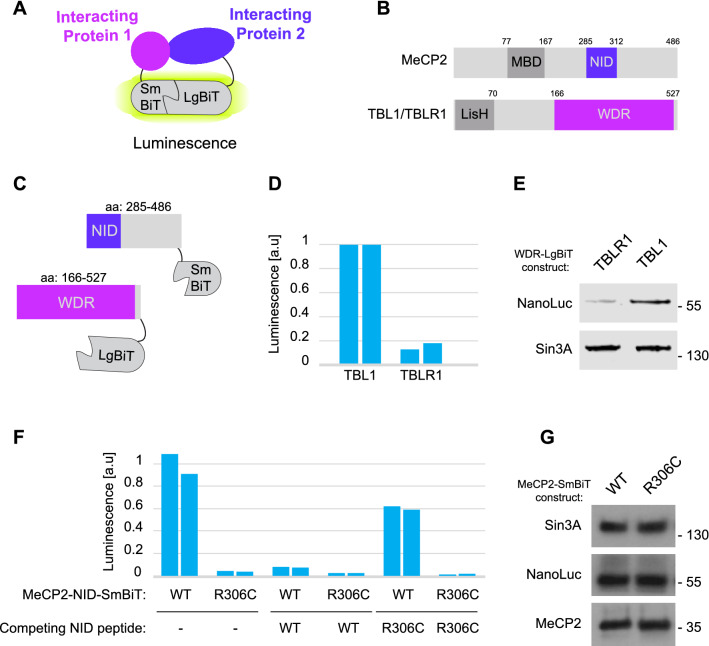
NanoLuc based protein complementation assay for MeCP2-TBL1 binding. (**A**) Schematic showing interaction between two proteins of interest bridging the large (LgBiT) and small (SmBiT) fragments of NanoLuc resulting in luminescence. (**B**) Functional domains of MeCP2 and TBL1/TBLR1. The MBD (methyl-CpG binding domain) is the DNA binding domain of MeCP2. The NID of MeCP2 mediates binding to the NCoR complex via an interaction with TBL1/TBLR1. The WD repeat (WDR) domain of TBL1/TBLR1 binds to the NID of MeCP2. The Lis homology (LisH) domain of TBL1/TBLR1 mediates self-association and incorporation into the NCoR complex. (**C**) Illustration of the MeCP2-SmBiT and TBL1/TBLR1-LgBiT fusion proteins used in this study. SmBiT is fused to a C-terminal fragment of MeCP2 (residues 285–486). LgBiT is fused to the WD repeat (WDR) domain of TBL1/TBLR1 (residues 166–527 for TBL1). (**D**) Robust luminescence is observed using extracts from cells transiently expressing MeCP2-SmBiT and TBL1/TBLR1-LgBiT. The two bars represent the results of biological replicates (n = 2) for each condition. (**E**) Western blotting with antibodies against NanoLuc and Sin3A (loading control) reveals stronger expression of the TBL1-LgBiT fusion protein than of TBLR1-LgBiT. (**F**) Luminescence observed with extracts from cells co-expressing MeCP2-SmBiT and TBL1-LgBiT is strongly reduced by introducing the R306C mutation into the NID of MeCP2. Luminescence is also inhibited by the addition of 20 µM of a peptide corresponding to the NID of MeCP2. A similar peptide carrying the R306C mutation has a much reduced inhibitory effect. The two bars represent the results of biological replicates (n = 2) for each condition. (**G**) Western blotting for MeCP2, NanoLuc and Sin3A (loading control) shows that the R306C mutation in MeCP2 does not affect the expression levels of the MeCP2-SmBiT and TBL1-LgBiT fusion proteins.

To facilitate the discovery of inhibitors of the interaction between MeCP2 and TBL1/TBLR1, we set out to devise a simple and scalable biochemical assay for measuring the interaction between these two proteins. We initially explored the possibility of screening compound libraries using a previously established fluorescence polarization assay that depends on purified recombinant TBL1/TBLR1^26^. However, our efforts were unsuccessful due to a combination of the relatively low binding affinity between an MeCP2-derived peptide and TBL1/TBLR1 (> 10 µM), and the limited yields of recombinant TBL1/TBLR1 obtained using bacterial and insect cell expression systems. We therefore applied an alternative approach based on a protein-fragment complementation assay in which two fragments of the luciferase NanoLuc are fused to interacting partner proteins^37,38^. The resulting luminescence signal depends on the affinity between the two interacting proteins of interest (Fig. 1A). This method was originally developed to allow for the interrogation of protein–protein interactions in vivo. However, in order to facilitate large scale screening of chemical libraries, we adapted the assay for use as a cell-free system.

We fused the large N-terminal fragment of NanoLuc (LgBiT) to the C-terminus of the WD repeat domains of both TBL1 and TBLR1, and the small C-terminal fragment of NanoLuc (SmBiT) to the C-terminus of the C-terminal half of MeCP2 (Fig. 1B and C). This region of MeCP2 contains the NID (NCoR interaction domain)—the motif in MeCP2 that binds to TBL1/TBLR1. Following expression of these fusion proteins in HEK293T cells, we detected a robust luminescence signal in lysates from these cells upon addition of the NanoLuc substrate furimazine. When expressed with MeCP2-SmBiT, we obtained an approximately ~ sixfold stronger luminescence signal using TBL1-LgBiT than with TBLR1-LgBiT (Fig. 1D). Western blotting with an antibody against NanoLuc revealed that this was likely due to stronger expression of the TBL1-LgBiT fusion protein when compared with TBLR1-LgBiT (Fig. 1E, Supplementary Fig. S1). Given the high degree of sequence conservation between the WD repeat domains of TBL1 and TBLR1 (92% sequence identity), particularly at the interface with MeCP2^26^, inhibitors of the interaction between MeCP2 and TBL1 would also be expected to block MeCP2 binding to TBLR1. We therefore decided to focus our efforts on the assay using TBL1.

To test the specificity of the assay, we performed control experiments using the RTT-causing R306C mutation within the NID of MeCP2. This single amino acid substitution, which is known to destroy the interaction between MeCP2 and TBL1/TBLR1^25,26^, resulted in a ~ 30-fold reduction in the observed luminescence signal (Fig. 1F). Western blotting using antibodies directed against NanoLuc and against MeCP2 showed that the R306C mutation did not affect the expression levels of either of the fusion proteins (Fig. 1G, Supplementary Fig. S1). As a final stage in characterization of the assay, we investigated the response to competitive inhibition of MeCP2-TBL1 binding by an MeCP2-derived peptide. Addition of a 35 amino acid peptide containing the TBL1/TBLR1 interaction motif from MeCP2 (amino acids 285-319) strongly inhibited the observed luminescence signal, whereas a corresponding control peptide with the R306C mutation failed to substantially affect the measured signal (Fig. 1F). Collectively, these observations demonstrate the specificity of the NanoLuc assay, and also the ability of this assay to detect specific competitive inhibitors of the MeCP2-TBL1 interaction.

### Pilot screen of a protein–protein interaction inhibitor compound collection

**Figure 2:**
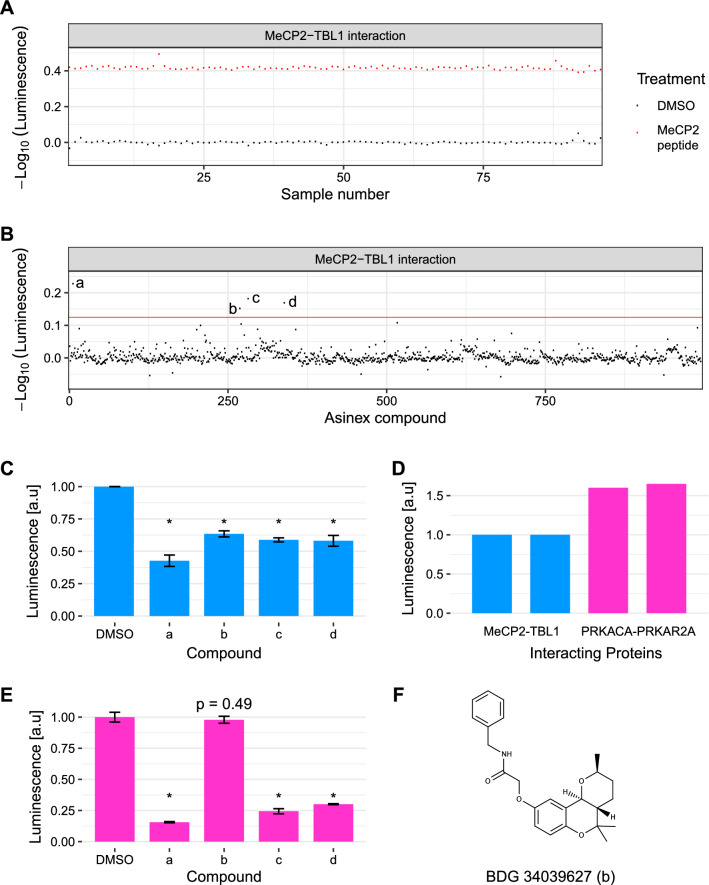
Screening for MeCP2-TBL1 inhibitors in a protein–protein interaction inhibitor library. (**A**) Z-factor determination using a short sequence-optimized MeCP2 peptide (MeCP2 K304Y, residues 298–309 of MeCP2) (red) at 50 μM as a positive control and DMSO (black) as a negative control. The measured luminescence for each sample was normalized to the mean luminescence observed with DMSO. The y-axis shows the -log_10_ of these normalized values. (**B**) Distribution of hits from a screen of 995 compounds from a custom Asinex protein–protein interaction inhibitor library assayed at 20 µM. The interaction between MeCP2 and TBL1 was monitored via the luminescence signal in a NanoLuc complementation assay. Measured luminescence for each compound was normalized to the mean luminescence value obtained with DMSO. The y-axis shows the − log_10_ of these normalized values. Four compounds (labelled a–d) show greater than 25% inhibition (threshold indicated by the red line). (**C**) Individual validation of the top four hits (n = 3; * p < 0.005 (t-test)). Error bars represent standard deviations. For all four compounds the degree of inhibition achieved at 20 µM was consistent with that observed in the original screen. (**D**) Robust luminescence observed using extracts from cells transiently expressing NanoLuc fragments as fusion proteins with two subunits of protein kinase A (SmBiT-PRKACA and LgBiT-PRKAR2A). The signal was approximately 1.6-fold greater than is observed using the MeCP2-TBL1 interaction assay. The two bars represent the results of biological replicates (n = 2) for each condition. (**E**) Specificity of primary Asinex library hits in the PKA control assay (n = 3; * p < 0.005 (t-test)). Error bars represent standard deviations. Only compound BDG 34039627 (b) did not affect luminescence in the PKA control assay. (**F**) Chemical structure of BDG 34039627.

The Z-factor is a statistic frequently used to assess the suitability of an assay for high throughput screening^39^. We calculated the Z-factor of our assay in 384-well plate format using a short 12 residue MeCP2 NID derived-peptide variant as positive control and DMSO as negative control^39^. This peptide harbours a lysine to tyrosine substitution at position 304 (K304Y) in the TBL1/TBLR1 interaction motif. The K304Y mutant peptide was identified as a stronger TBL1/TBLR1 binder using a peptide SPOT array constructed to introduce all single amino acid substitutions in the residues of MeCP2 that make direct contacts with TBL1/TBLR1 (Supplementary Fig. S2). The mutant peptide was able to compete for MeCP2-TBL1 binding with an IC_50_ of ~ 40 μM as measured in the NanoLuc assay, and showed reproducible MeCP2-TBL1 inhibition at 1-, 5- and tenfold extract dilutions (Supplementary Fig. S2). Overall, the assay showed an excellent separation between the positive and negative controls, and low signal variance with a calculated Z-factor of 0.85 (Fig. 2A)^39^.

We next performed a pilot screen using a custom library of 995 compounds comprised of a structurally diverse subset selected from the Asinex protein–protein interaction library^40^. Initial screening identified four compounds that inhibited the luminescence signal obtained in the MeCP2-TBL1 assay by at least 25% when applying a robust strictly standardized mean difference (SSMD*) threshold score of ≥ 3 for hit selection^41^ (Fig. 2B, Table S1). Two of the identified compounds display a high degree of structural similarity (Supplementary Fig. S3, compounds a and c). In order to validate the results obtained from the pilot survey, we tested these four compounds individually in a second independent experiment. Consistent with the screening data, we found that all four compounds reduced the luminescence signal obtained in the MeCP2-TBL1 NanoLuc assay by approximately 40–60% (Fig. 2C).

### Identification of false positives via a NanoLuc-based counter screen

The MeCP2-TBL1 assay does not distinguish between *bona fide* inhibitors of the MeCP2-TBL1 interaction and molecules that non-specifically interfere with the assay, such as inhibitors of the NanoLuc active site^42^, inhibitors of the binding interface between the two NanoLuc fragments or compounds that interfere with the generation of luminescence signals by other means. To distinguish such false positives from genuine inhibitors of the interaction between MeCP2 and TBL1/TBLR1, a control screen is essential, utilising the same NanoLuc SmBiT and LgBiT fragments, but with an unrelated protein interaction pair. The interaction between PRKACA and PRKAR2A, which are the catalytic and regulatory subunits of human protein kinase A (PKA) respectively, has previously been measured using a NanoLuc complementation assay^38^. We tested this pair of interacting proteins for activity in a NanoLuc complementation assay under our conditions. When using PRKACA and PRKAR2A, we observed a strong luminescence signal around 1.6-fold greater than obtained using the interaction between MeCP2 and TBL1 (Fig. 2D). We therefore selected the PKA-based NanoLuc complementation as a suitable control assay. The effects of the four compounds identified from the Asinex library were assessed in the control PKA-based NanoLuc assay. Three of the four compounds, including the two structurally related compounds, caused a significant reduction in observed luminescence and, hence, were false positives that interfere with NanoLuc luminescence (Fig. 2E, Supplementary Fig. S3). However, compound BDG 34039627 (b) did not cause a reduction in luminescence and is therefore a *bona fide* candidate inhibitor of the MeCP2-TBL1 interaction (Fig. 2F).

### A MeCP2-TBL1 inhibitor drug repurposing screen of FDA-approved compounds

**Figure 3:**
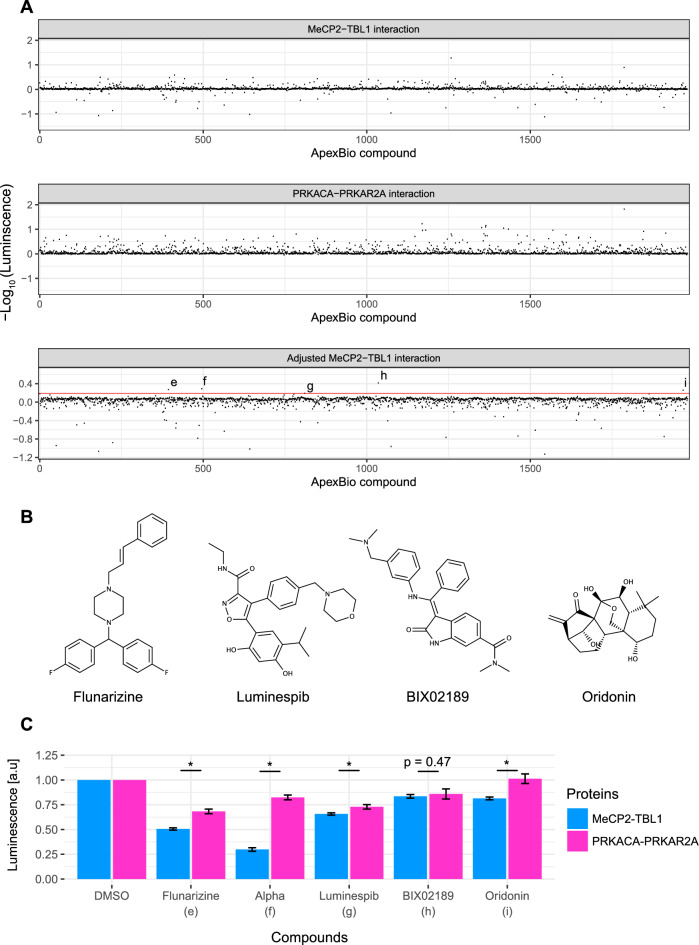
Screening of an FDA-approved drug library with the MeCP2-TBL1 assay and PRKACA-PRKAR2A counter assay. (**A**) Distribution of hits from a screen of 1981 compounds from the ApexBio FDA-approved Drug Library assayed at 20 µM. The interaction between MeCP2 and TBL1 was monitored via the luminescence signal in a NanoLuc complementation assay (top panel). Also shown is the distribution of hits from a control screen using a NanoLuc complementation assay based on the interaction between PRKACA and PRKAR2A (middle panel). For the top and middle panels the measured luminescence for each compound was normalized to the mean luminescence obtained with DMSO. The y-axis shows the − log_10_ of these normalized values. The bottom panel shows inhibition in the MeCP2-TBL1 assay after adjusting for activity of compounds in the PRKACA-PRKAR2A assay. The top five hits (e–i) are indicated above the 35% inhibition threshold (red line). (**B**) Chemical structures of the non-peptide hits from dual screening of the ApexBio library with the MeCP2-TBL1 assay and the control PRKACA-PRKAR2A assay. (**C**) Individual validation of the top five hits from the ApexBio library screen against both the MeCP2-TBL1 and the PRKACA-PRKAR2A NanoLuc complementation assays. At 20 µM, flunarizine, luminespib, oridonin, and the alpha-1 antitrypsin fragment 235–243 all achieved statistically significantly greater inhibition in the MeCP2-TBL1 assay than in the control assay (n = 7; * p < 0.001 (t-test)). Error bars represent standard deviations. BIX02189 did not have a statistically different effect between the two assays (n = 7; p = 0.47 (t-test)).

Drug repurposing, in which approved drugs are screened against a target of interest, is a potentially timesaving and cost-efficient approach that can de-risk the development of small molecule-based therapies for new disease indications^43^. We therefore screened a library of 1981 FDA-approved drugs and other compounds at various stages of clinical research against the MeCP2-TBL1 interaction assay, and also against the PKA-based control assay in a counter-screen. Both screens identified many apparent weak interaction inhibitors (Fig. 3A, upper and middle panels, Table S2). Inspection of the two datasets together, however, revealed that many molecules that apparently inhibited MeCP2-TBL1 binding were also active as inhibitors in the control assay, suggestive of non-specific effects on NanoLuc activity. To account for these non-specific effects, we used the degree of inhibition observed for each compound in the control assay to normalize the degree of inhibition observed with the MeCP2-TBL1 interaction. Analysing the data in this way, and setting a threshold of SSMD* ≥ 3 (~ 35% inhibition), revealed five candidate inhibitors of the MeCP2-TBL1 interaction (Fig. 3A, lower panel). These compounds were flunarizine, oridonin, BIX02189, luminespib, and the alpha-1 antitrypsin fragment 235–243 (AAT) (Fig. 3B).

### Validation of candidate inhibitors of MeCP2-TBL1 binding

To verify the results of this second pilot screen, we tested the five candidate inhibitors of the MeCP2-TBL1 interaction individually in both assays. For four out of the five compounds (flunarizine, luminespib, oridonin, and the alpha-1 antitrypsin fragment 235-243), we detected inhibition of the luminescence signal in the MeCP2-TBL1 assay that was statistically significantly greater than any inhibition observed in the control PKA-based assay (Fig. 3C). To determine the potency of the primary hits from both pilot screens, MeCP2-TBL1 extracts were incubated with different concentrations (0.3–60 μM) of each of the putative inhibitors and the luminescence signal was measured. Half-maximal inhibitory concentrations (IC_50_) were calculated from the inhibition curves and specificity was determined by running the control PKA-based assay in parallel (Supplementary Fig. S4). AAT and BDG 34039627 showed greatest potency with IC_50_ of 6 μM and 18 μM, respectively. Both were able to return the luminesce signal back to baseline within the tested concentration range, and both showed a clear separation between MeCP2-TBL1 inhibition and inhibition of the PKA-based control. Although less potent, flunarizine (IC_50_ 24 μM) and luminespib (IC_50_ 31 μM) also showed a clear separation between MeCP2-TBL1 inhibition and inhibition of the PKA control, but without being able to completely inhibit MeCP2-TBL1 binding within the tested concentration range. Finally, BIX02189 (IC_50_ 28 μM) and oridonin (IC_50_ 34 μM) showed only modest selectivity for MeCP-TBL1 inhibition over PKA assay inhibition. Although the degree of inhibition was modest for all the identified MeCP2-TBL1 interaction inhibitors, the chemical structures of BDG 34039627, flunarizine and luminespib might form the basis of screening a larger set of related analogs using the same screening methodology. Importantly, these results demonstrate that our dual screening method is a powerful approach to identifying specific inhibitors of the MeCP2-TBL1 interaction from large compound libraries.

## Discussion

In recent years, huge effort has been devoted to studying the genetic basis of neurodevelopmental disorders. Exome sequencing of tens of thousands of affected individuals has identified numerous genes that are mutated in these conditions^44–46^. Despite this progress, understanding of the molecular mechanisms perturbed in many of these disorders remains sparse, and even in cases where detailed understanding is available, such knowledge has not been translated into pharmacological interventions. MDS is an example of a disorder where much has been learned about the underlying molecular pathology, but without new therapies becoming available to patients. Knowledge that the toxicity of excess MeCP2 in MDS is dependent on the ability of MeCP2 to interact with TBL1/TBLR1^29,30^ raises the possibility of pharmacological disruption of the MeCP2-TBL1/TBLR1 interaction as a therapeutic strategy for MDS. This work describes our efforts to translate this progress in basic biology into a meaningful basis for clinical intervention by identifying small molecule inhibitors of the MeCP2-TBL1 interaction.

We developed a NanoLuc complementation assay to screen for inhibitors of the MeCP2-TBL1 interaction. An important aspect of the work was to optimize the assay for use as an in vitro cell-free system, which can be readily applied to high-throughput screening of compound libraries. Another key feature of the approach is the use of a control NanoLuc complementation assay based on the interaction between the subunits of PKA. This allows the identification and rejection of false positives arising due to non-specific effects of compounds on NanoLuc activity. Other intrinsic advantages of the NanoLuc approach include its simple luminescence read-out, sensitive detection of relatively low affinity interactions, and use of crude cell extracts without the need for purified recombinant proteins. Using this combination of methods, we have screened almost 3000 compounds and uncovered six candidate inhibitors of the MeCP2-TBL1 interaction. Importantly, our ability to identify these compounds demonstrates the future feasibility of screening larger libraries for more potent inhibitors of the MeCP2-TBL1 interaction. Of the six candidate inhibitors, BDG 34039627, AAT, flunarizine and luminespib all showed appreciable specificity for the MeCP2-TBL1 interaction. However, these compounds only impinge upon the MeCP2-TBL1 interaction at relatively high concentrations (IC_50_ > 10 µM), and so would require optimization before progression to in vivo preclinical studies. AAT is a linear peptide and is therefore of limited interest due to the liabilities of peptides for drug development. Structure–activity relationship (SAR) analysis of commercially available BDG 34039627, flunarizine and luminespib analogs might identify more potent inhibitors and form the starting point to develop optimized MeCP2-TBL1 inhibitors.

Progressing potent MeCP2-TBL1 interaction inhibitors towards use in the clinic will require significant further research. For example, it will be necessary to determine whether such interaction inhibitors are effective against intact full-length proteins both in vitro and in vivo. This will be facilitated by the availability of several different assays for measuring the interaction between MeCP2 and TBL1^25,26^. Much pre-clinical work will also be needed to test whether MeCP2-TBL1 interaction inhibitors can indeed ameliorate the pathology associated with MDS. The previously reported cellular and animal models^29–31^ of MDS should be extremely useful for this research.

There are potential caveats and limitations associated with our strategy of treating MDS using small molecules to disrupt the interaction between MeCP2 and TBL1/TBLR1. Effective therapy would require regular administration of a drug that is able to cross the blood–brain barrier, and since MeCP2 loss-of-function is associated with RTT, the therapeutic window may be narrow. The strategy also assumes that it will be possible to block MeCP2 binding to TBL1/TBLR1 without significantly disrupting other essential functions of TBL1/TBLR1. While it is clear that the MeCP2 binding WDR domains of TBL1/TBLR1 are not required for incorporation of these proteins into the NCoR co-repressor complex, the molecular functions of TBL1/TBLR1 remain poorly understood. Therefore critical MeCP2-independent functions for their WDR domains cannot be excluded.

Using small molecules to inhibit the interaction between MeCP2 and TBL1/TBLR1 is not the only plausible approach to developing a therapy for MDS. As MDS is a gain-of-function disorder, a variety of methods aimed at inhibiting MeCP2 represent potential treatment strategies. For example, antisense oligonucleotides have been used to modulate MeCP2 expression levels in a mouse model of MDS^31^. A CRISPR-based approach has also been used to normalize MeCP2 copy number in a primary cell line derived from an individual with MDS^47^. However, delivery of either antisense oligonucleotides or CRISPR/Cas9 enzymes to the human central nervous system will likely be challenging. Others have developed inhibitors of the DNA-binding methyl-CpG binding domain (MBD) of MBD2^48^. The development of analogous DNA binding inhibitors targeting the MBD of MeCP2 could also be a useful way of modulating MeCP2 function in MDS^30^. The diversity of approaches adopted by different research groups in this area allows room for optimism, since success could be achieved by one or more of these methods working either individually or in combination. This is reinforced by the fact that, unlike for many other drug targets, success would only require a modest reduction in the MeCP2-TBL1/TBLR1 interaction. The prospect of a therapy for MDS is not only exciting clinically – it would also answer fundamental biological questions about the degree of reversibility of the damage done to the human brain in a neurodevelopmental disorder.

## Methods

### Expression vectors

The human MeCP2 coding sequence (residues 285-486) was cloned between the HindIII and AgeI restriction sites of pEGFP-N1 (Clontech). The AgeI-NotI restriction fragment (encoding EGFP) was then replaced with a codon optimized SmBiT sequence to yield the MeCP2-SmBiT expression plasmid. The TBL1-LgBiT expression vector was derived from a TBL1-mCherry plasmid^25^ by replacing the AgeI-NotI restriction fragment (encoding mCherry) with a codon optimized LgBiT sequence. SmBiT-PRKACA and LgBiT-PRKAR2A plasmids are commercially available (Promega, N203 and N204).

### Antibodies and peptides

Western blotting was performed using antibodies against NanoLuc (Promega), MeCP2 (Sigma, M6818) and Sin3A (Abcam, Ab3479). The 35 amino acid MeCP2 wild-type and R306C NID peptides (residues 285-319) were the same as described previously^25^ and were used at a concentration of 20 µM in the competition assays. The biotin at the N-terminus of these peptides was not relevant to the experiments in this work. The SPOT peptide array had peptides based on mouse MeCP2 residues 297–308 (HETVLPIKKRKT). Residues 302–305 (PIKK) – which make direct contact with TBLR1—were systematically altered to each of the 20 naturally occurring amino acids^49^. The array membrane was incubated with recombinant HIS_6_‐tagged TBLR1 (2 µM) and immunodetection was performed with anti-HIS antibody (Sigma, H1029). Uniform synthesis efficiency across the array was ascertained by UV absorption. The higher affinity TBL1/TBLR1 binding mutant (K304Y) was incorporated into a 12 amino acid peptide (Ac-ETVLPIYKRKTR-NH_2_) corresponding to residues 298-309 of MeCP2 which was designated UMT026-2. Note the shorter N-terminus and longer C-terminus of this peptide compared to the sequences used on the SPOT array. UMT026-2 was used at 50 µM when calculating the Z-factor^39^.

### Cell culture

HEK293 cells were grown in DMEM (Wisent, 319-005-CL) supplemented with 10% fetal calf serum (Sigma, F1051) at 37 °C under 5% CO_2_. Cells were seeded into 15 cm plates and co-transfected with NanoLuc plasmids using linear polyethylenimine 25 kDa (PEI 25 K; Polysciences, 23966). Cells were harvested after 24 h and cell pellets were stored at − 80 °C.

### NanoLuc assays

Lysis of cells from a single plate was performed by resuspending frozen cells in 20 ml passive lysis buffer (Promega, E1941) supplemented with 20 µg/ml RNase A (Qiagen, 19101). A single 15 cm plate of cells typically yielded sufficient extract for up to 12.5 384-well assay plates and extracts remained stable and active for at least 2 h at room temperature. In the screens described here, the lysate obtained from a single 15 cm plate was used for 2.5 384-well assay plates. NanoLuc substrate (furimazine) (Promega, N1110) was diluted 1000-fold in passive lysis buffer (Promega, E1941) and added 1:1 to the lysate. Screening libraries were purchased from Asinex^40^ and ApexBio (FDA-approved Drug Library, L1021). Compounds were dispensed using an Echo acoustic dispenser and tested at a final concentration of 20 µM in a total assay volume of 20 μl in 384-well plate format. DMSO was added to each well at a final concentration of 0.2% (v/v) and wells containing only DMSO were used as negative control. After 30 min incubation at room temperature, luminescence was measured on a Tecan M1000 multi-mode plate reader. Raw luminescence reads from each well on each plate were normalized to the median luminescence signal for that plate. In the ApexBio screen, the scaled and normalized PKA signal for each compound was subtracted from the MeCP2-TBL1 signal for the same compound. After plate normalization the robust SSMD (SSMD*) was calculated as described elsewhere^50^. A threshold of SSMD* ≥ 3 was applied for discerning primary hits.

### Determination of half-maximal inhibitory concentrations

Assays were performed as described above except instead of a single concentration, a threefold serial dilution ranging from 60 to 0.03 μM was tested in triplicate for each of the compounds. Half-maximal inhibitory concentrations were calculated using global nonlinear regression by fitting the response data sets of all compounds simultaneously with a four-parameter logistic curve of log inhibitor concentration versus normalized luminescence using GraphPad Prism. During fitting, the maximum and minimum curve values were shared between the data sets. The luminescence signal of each compound concentration was normalized by dividing the luminescence signal of the DMSO control.

## Supplementary Information

Supplementary Figures.

Supplementary Table S1.

Supplementary Table S2.

Supplementary Legends.

## Data Availability

All data generated or analysed during this study are included in this published article and its supplementary information files.
