# Supplementary figures and images for: A screen for MeCP2-TBL1 interaction inhibitors using a luminescence-based assay
Source: Sci Rep. 2023 Mar 8;13:3868. doi: 10.1038/s41598-023-29915-z (PMC9995496; doi:10.1038/s41598-023-29915-z)

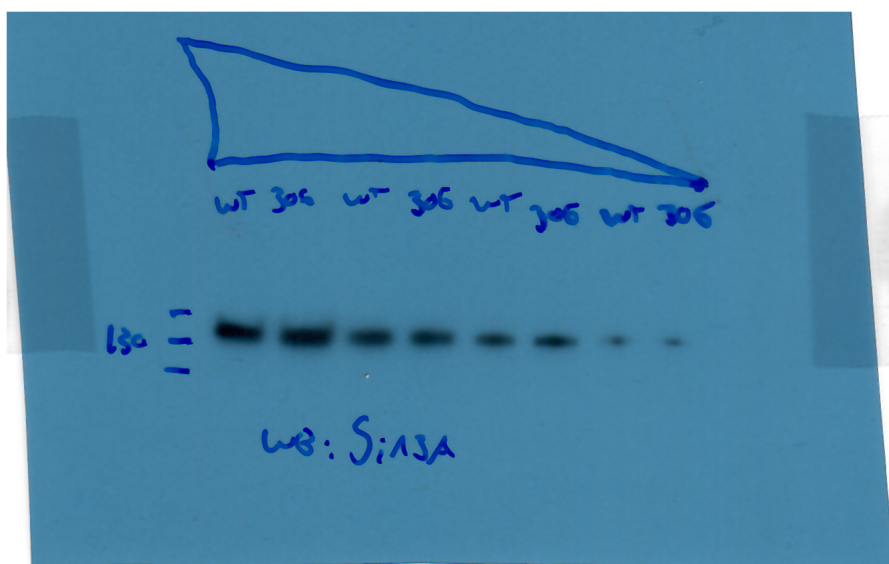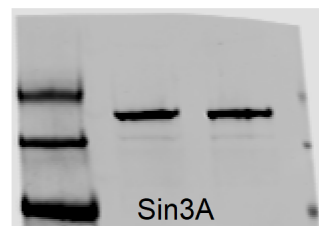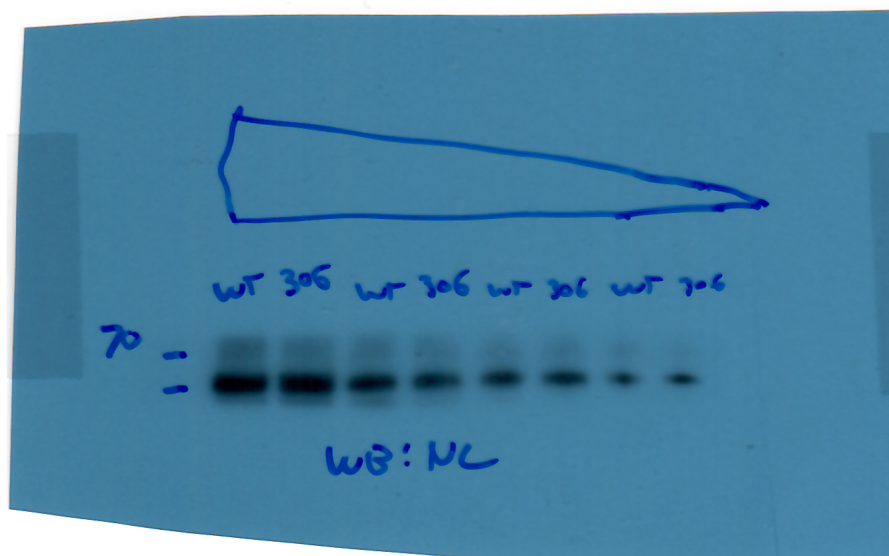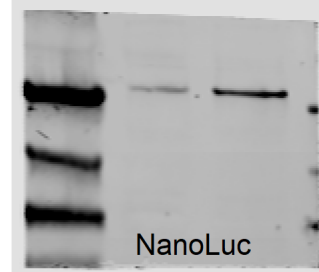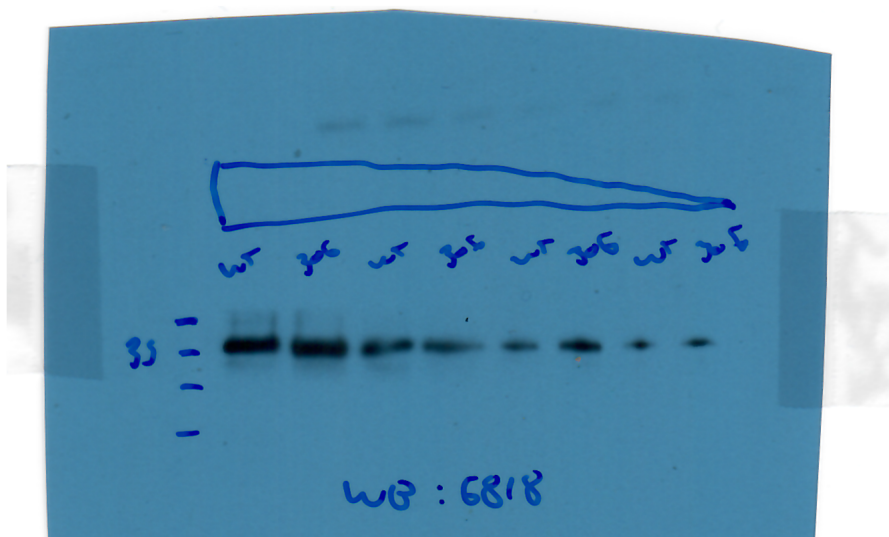

Figure S1

**A**

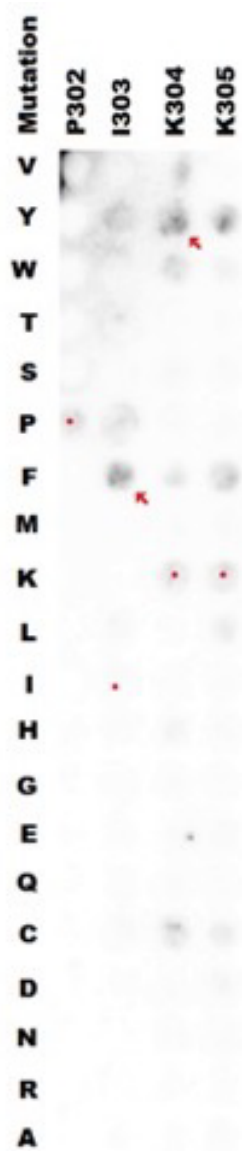

**B**

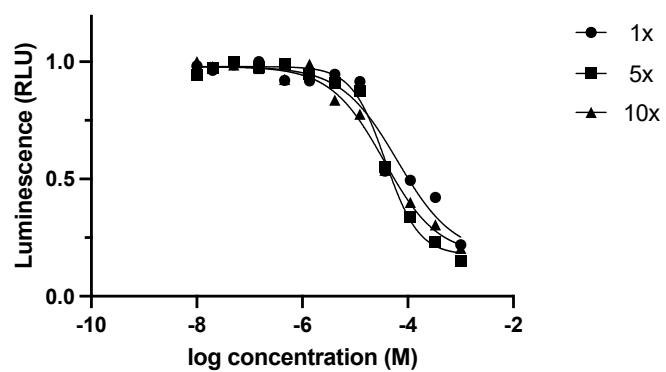

Figure S2

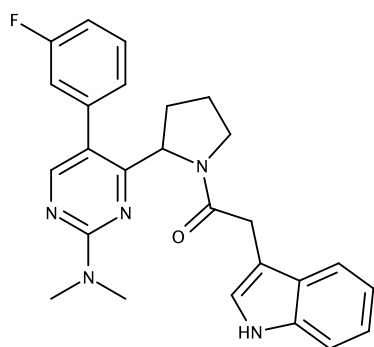

BDE 26723608 (a)

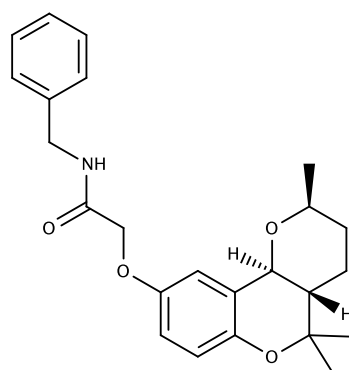

BDG 34039627 (b)

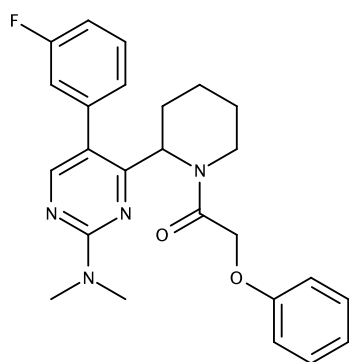

BDE 26725534 (c)

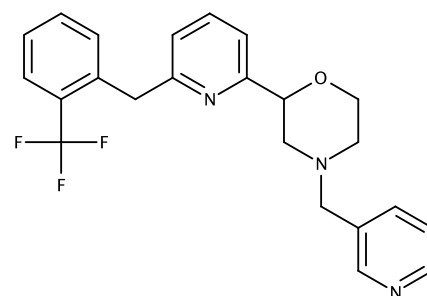

BDF 27574784 (d)

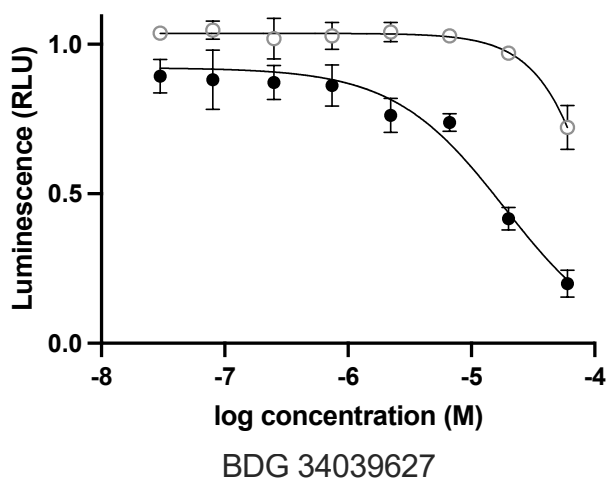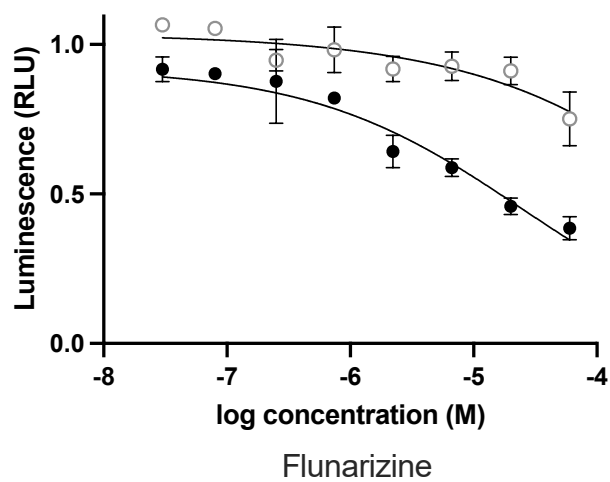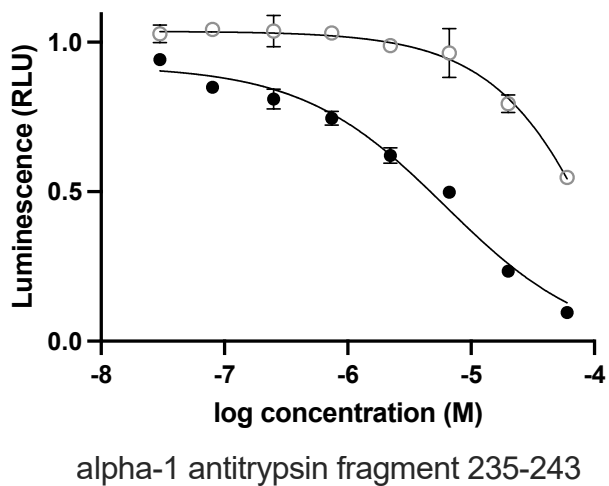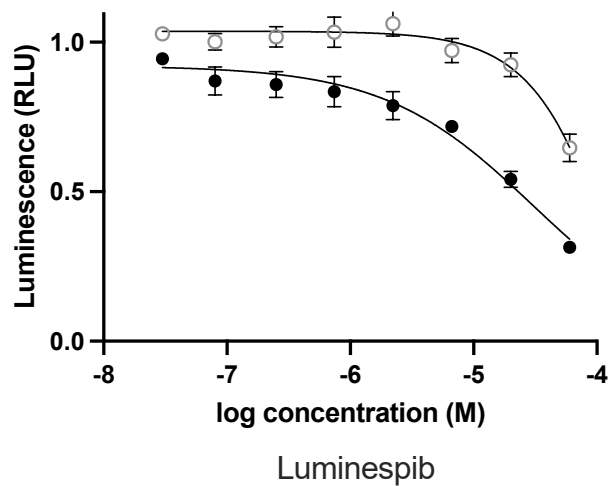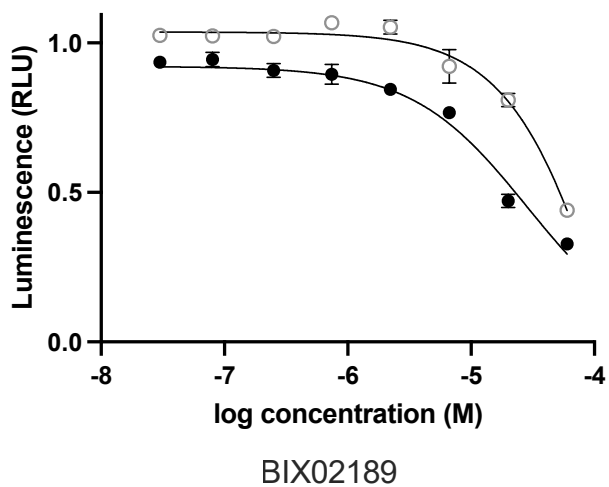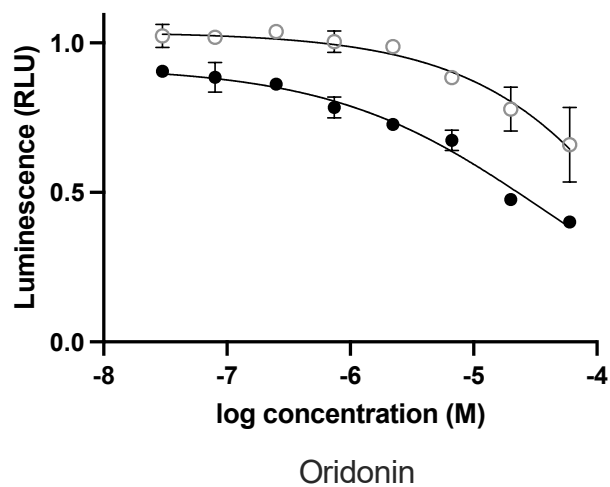

Figure S4

Supplement: Supplementary file 1 — Supplementary Figures. [file 41598_2023_29915_MOESM1_ESM.pdf]
