# Supplementary material for: A screen for MeCP2-TBL1 interaction inhibitors using a luminescence-based assay
Source: Sci Rep. 2023 Mar 8;13:3868. doi: 10.1038/s41598-023-29915-z (PMC9995496; doi:10.1038/s41598-023-29915-z)
Supplement: Supplementary file 4 — Supplementary Legends. [file 41598_2023_29915_MOESM4_ESM.docx]

**Supplementary Figure S1: Uncropped Western blots**

Uncropped images corresponding to Western blots in figures 1E and 1G.

**Supplementary Figure S2: MeCP2 peptide SPOT array and validation**

**(A)** SPOT array of 12 amino acid peptides (residues 297-308 of mouse MeCP2) comprising all 20 natural amino acid substitutions in positions 302-305. The wild-type amino acids at these positions (Pro, Ile, Lys and Lys) make directs contacts with TBLR1. Arrows denote spots with increased binding and red dots indicate the wild-type amino acid. **(B)** In solution validation of the K304Y peptide (residues 298-309 of mouse MeCP2) in the MeCP2-TBL1 NanoLuc competition assay. The dose response curve of MeCP2 K304Y shows an IC_50_ of approximately 40 μM and reproducible MeCP2-TBL1 interaction inhibition at 1-, 5- and 10-fold extract dilutions.

**Supplementary Figure S3: Chemical structures of this hits from compound library screening**

Chemical structures of the top four hits from primary screening the Asinex library with the MeCP2-TBL1 NanoLuc assay. Two of these compounds (BDE26723608 and BDE26725534) displayed structural similarity.

**Supplementary Figure S4: IC_50_ determination for MeCP2-TBL1 candidate inhibitors**

Compounds were tested in triplicate at concentrations ranging from 0.3 to 60 μM against MeCP2-TBL1 extract (closed circles) or PKA control extract (open circles). Luminescence was normalized relative to the luminescence signal of a DMSO control.

**Supplementary Table S1**

Inhibition achieved by individual compounds in the Asinex library screen.

**Supplementary Table S2**

Inhibition achieved by individual compounds in the ApexBio library screen.
